# A Population of Langerin-Positive Dendritic Cells in Murine Peyer's Patches Involved in Sampling β-Glucan Microparticles

**DOI:** 10.1371/journal.pone.0091002

**Published:** 2014-03-14

**Authors:** Magdia De Jesus, Gary R. Ostroff, Stuart M. Levitz, Toni R. Bartling, Nicholas J. Mantis

**Affiliations:** 1 Division of Infectious Diseases, Wadsworth Center, New York State Department of Health, Albany, New York, United States of America; 2 Program in Molecular Medicine, University of Massachusetts Medical School, Worcester, Massachusetts, United States of America; 3 Department of Medicine, University of Massachusetts Medical School, Worcester, Massachusetts, United States of America; 4 College of Nanoscale Sciences and Engineering, State University of New York, Albany, New York, United States of America; 5 Department of Biomedical Sciences, University at Albany, Albany, New York, United States of America; The Ohio State University, United States of America

## Abstract

Glucan particles (GPs) are 2–4 μm hollow, porous shells composed of 1,3-β-D-glucan that have been effectively used for oral targeted–delivery of a wide range of payloads, including small molecules, siRNA, DNA, and protein antigens. While it has been demonstrated that the transepithelial transport of GPs is mediated by Peyer's patch M cells, the fate of the GPs once within gut-associated lymphoid tissue (GALT) is not known. Here we report that fluorescently labeled GPs administered to mice by gavage accumulate in CD11c^+^ DCs situated in Peyer's patch sub-epithelial dome (SED) regions. GPs appeared in DCs within minutes after gavage and remained within the SED for days afterwards. The co-administration or sequential administration of GPs with differentially labeled GPs or poly(lactic-co-glycolic acid) nanoparticles demonstrated that the SED DC subpopulation in question was capable of internalizing particles of different sizes and material compositions. Phenotypic analysis identified the GP-containing DCs as being CD8α^-^ and CD11b^lo/-^, suggesting they are the so-called myeloid and/or double negative (DN) subset(s) of PP DCs. A survey of C-type lectin receptors (CLRs) known to be expressed by leukocytes within the intestinal mucosa revealed that GP-containing SED DCs were positive for Langerin (CD207), a CLR with specificity for β-D-glucan and that has been shown to mediate the internalization of a wide range of microbial pathogens, including bacteria, viruses and fungi. The presence of Langerin^+^ DCs in the SED as determined by immunofluorescence was confirmed using Langerin E-GFP transgenic mice. In summary, our results demonstrate that following M cell-mediated transepithelial transport, GPs (and other micro/nanoparticles) are sampled by a population of SED DCs distinguished from other Peyer's patch DC subsets by their expression of Langerin. Future studies will be aimed at defining the role of Langerin in antigen sampling and antigen presentation within the context of the GALT.

## Introduction

While the oral route remains the most desirable means by which to administer mucosal vaccines, the poor efficacy of antigen uptake into the gut-associated lymphoid tissues remains a major problem. β-1,3-D-glucan particles (GPs) are non-toxic polysaccharide shells (2–4 μm diameter) derived from the cell walls of *Saccharomyces cerevisiae* [1]. GPs have been successfully synthesized and loaded with an array of soluble payloads, including proteins, DNA, siRNA, and nanoparticles and delivered to mice by a variety of routes [2], . Subcutaneous immunization of mice with ovalbumin (OVA)-loaded GPs induced strong OVA-specific Th1- and Th17-biased CD4^+^ responses, as well as antigen-specific serum IgG_1_ responses [2], [3]. *In vitro*, GP-OVA was >100-fold more potent than soluble OVA at stimulating the proliferation of OVA-reactive transgenic CD8^+^ OT-I and CD4^+^ OT-II T cells using dendritic cells (DCs) as antigen-presenting cells. Part of the effectiveness of GPs as vaccine delivery vehicles is due to the intrinsic adjuvant properties of β-1,3-D-glucans [7], [8], [9]. GPs are rapidly internalized by DCs and macrophages expressing Dectin-1 and complement receptors [2]. Dectin-1 agonists like β-glucans signal through Syk kinase and CARD9 to induce the production of IL-10 and IL-2 in DCs [8].

However, the use of GPs as a platform for the orally delivery of a variety of payloads is only just now being investigated. For example, Aouadi and colleagues recently demonstrated that the delivery of MAP4K siRNA-loaded GPs to mice by gavage resulted in the targeted suppression TNF-α mRNA in macrophages in the peritoneum, spleen, liver and lung, and lowered TNF-α levels in the serum. While Aouadi and colleagues highlighted the potential utility of GPs as an oral delivery vehicle that could be utilized to attenuate systemic and local inflammatory responses like Crohn's disease, it did not address the mechanism(s) by which GPs gained entry into the intestinal mucosa. Recently, however, De Smet and colleagues demonstrated using mouse ligated ileal loops that GPs are transported across intestinal epithelium by Peyer's patch M cells[10]. M cells are a specialized epithelial cell type found within the so-called follicle-associated epithelium (FAE) that overly gut-associated lymphoid tissues (GALT) like Peyer's patches (PP) and isolated lymphoid follicles (ILF) in the small and large intestines[11], [12]. In Peyer's patches, the so-called sub-epithelial dome (SED) region consists of a rich network DCs that are postulated to be involved in the active sampling of antigens following M cell transport [13], [14], [15], [16], [17].

In this study, we have investigated the fate of GPs following uptake into murine PP. We report that fluorescently labeled-GPs accumulate in a subset of SED DCs that are unique in that they express Langerin (CD207), a C-type lectin receptor (CLR) normally associated with non-mucosal DCs but that has been observed in the PP [18]. The subset of Langerin^+^ SED DCs we describe were also responsible for the uptake of fluorescently labeled poly(lactic-co-glycolic acid) (PLGA) nanoparticles following transport into the PP. As it is well-established that Langerin plays a role in pathogen recognition [19], [20], [21], we speculate that the Langerin^+^ SED DCs that we have identified in mouse PPs may play an important role in initiating and regulating mucosal immune responses to luminal antigens.

## Materials and Methods

### Mice

BALB/c female mice (8–12 weeks old) were obtained from Taconic Farms (Hudson, NY). C57BL/6 mice and *Lang-DTREGFP* (B6.129S2-Cd207tm3Mal/J) mice were obtained from the Jackson Laboratory (Bar Harbor, ME). Animals were housed under conventional, specific pathogen-free conditions and were treated in compliance with the Wadsworth Center's Institutional Animal Care and Use Committee (IACUC) guidelines. Dectin-1 knockout, Mannose Receptor (MR) knockout and Dectin-1-MR double-knockout mice were crossed bred and housed at the University of Massachusetts Medical School under conventional, specific pathogen-free conditions and were treated in compliance with University of Massachusetts IACUC guidelines. Dectin-1 and MR knockout mice were gifts of Gordon Brown and Michel Nussenzweig (Rockefeller University), respectively.

### Ethics statement

Experiments described in this study that involve mice were reviewed and approved by the Wadsworth Center's Institutional Animal Care and Use Committee (IACUC) under protocol #12–428. The Wadsworth Center complies with the Public Health Service Policy on Humane Care and Use of Laboratory Animals and was issues assurance number A3183-01. Moreover, the Wadsworth Center is fully accredited by the Association for Assessment and Accreditation of Laboratory Animal Care (AAALAC). Obtaining this voluntary accreditation status reflects that Wadsworth Center's Animal Care and Use Program meets all of the standards required by law, and goes beyond the standards as it strives to achieve excellence in animal care and use.

### GPs and PLGA nanoparticles

GPs were derived from *S. cerevisiae* (Fleischmann's baker's yeast) using a series of alkaline and acidic extraction steps as previously described [2], [3], [4], [5], [6]. GPs were labeled with dichlorotriazinylaminofluorescein (DTAF) (Sigma Aldrich, St. Louis, MO) or Alexa-Fluor 633 (Invitrogen, Grand Island, NY) in 0.1 M borate buffer pH 8.5 overnight at room temperature (RT). Labeling reaction was quenched in 1 M Tris base for 30 minutes at RT. GPs were washed until excess dye was no longer present and supernatants were clear. GPs were then sterilized in 70% ethanol for 30 min at RT in the dark followed by washing in sterile PBS and stored at −20°C Texas Red-labeled poly(lactic-co-glycolic acid) (PLGA) particles were a gift of Dr. Nejat Elgimez (University of Louisville, Louisville, KY).

### Oral gavage and tissue collection

Mice were gavaged using a 22 G×1.5-in. blunt-end feeding needle (Popper Scientific, New Hyde Park, NY). Fifteen minutes prior to gavage with GPs or PLGAs, mice received 10 mM N-acetyl cysteine (Sigma, St. Louis MO) to temporarily dissociate mucus in the intestinal lumen. GPs (1×10^7^ total) were gavaged in 200 μl of 0.1 M sodium bicarbonate. PPs were harvested at time points ranging from 5 min to 72 hrs.

### PP cryosectioning and immunostaining

PPs were harvested and cryosectioned as described [22]. For immunostaining, cryosections were immersed in acetone for 2 minutes and then washed three times with PBS-Tween 20 (PBS-T; 0.05%, vol/vol). Slides were carefully dried and tissue sections were encircled with an ImmEdge hydrophobic pen (Vector labs, Burlingame CA). Sections were blocked with 2% goat serum in PBS for 30 min at 37°C followed by a brief wash in PBS and then 10 min at 37°C in spent supernatants of ATCC 2.4.G2 cells as means to block FcγRII receptors. Primary and secondary antibodies used in this study are described in **Table S1**. Primary antibodies were incubated on tissues sections for 1 hr at 37°C in a moisture chamber. Sections were washed then with PBS-T before being incubated with labeled relevant secondary antibodies. To preserve the sections for up to a week, they were post fixed with 4% paraformaldehyde for 4 min at RT followed by a wash PBS-T. Sections were mounted with a glass coverslip and sealed with ProLong Gold antifade reagent (Invitrogen, Grand Island, NY). Images were captured using a Leica SP5 confocal laser scanning microscope (Leica, Wetzlar, Germany) and processed using Fiji Software [23]. Montages of confocal images were generated using Adobe Photoshop version 13.0×32 (Adobe Systems, San Jose CA).

### Isolation of PP cells for flow cytometry, imaging flow cytometry and DC purification

Single cell suspensions of PP cells were isolated as described previously [22]. For flow cytometry, isolated cells were transferred to a 96 round bottom well plate and subjected to centrifugation at 1000 × g for 5 min. Fc receptors were blocked for 15 min with supernatants from the ATCC 2.4.G2 cell line before the addition of the primary antibody cocktail (20 ug/ml). Cells were incubated on ice for 30 min with continuous rocking and then washed and fixed in 60 mM PIPES, 25 mM HEPES, 10 mM EGTA, 4 mM MgCl_2_ at pH 6 (PHEM) buffer containing 1% paraformaldehyde and subjected to flow cytometry using a FACS Calibur. Results were analyzed using Cell Quest Pro software version 5.2. For imaging flow cytometry was performed using the ImageStream (Amnis, Seattle WA) equipped with a 480–560 laser. Cells (1×10^6^) were prepared as a 50 μl volume in PHEM buffer containing 1% paraformaldehyde. For DC purification, total PP cells were subjected to CD11c^+^ Easy Sep Positive Selection Kit (Stem Cell Technologies, Vancouver, BC), according to manufacturer's instructions.

### Preparation of cDNA and RT-PCR

RNA was isolated from total PP or PP CD11c^+^ cells using the RNeasy RNA Isolation Kit (Qiagen, Valencia CA) and the Turbo DNA-free kit (Ambion, Applied Biosystems). DNA-free, total RNA (50 ng/μl) was used to make cDNA, by incubating with 50 μM random oligo dTprimers for 5 min at 65°C. The mixture was then cooled for 5 min at room temperature and the Superscript III first strand synthesis kit (Invitrogen, Grand Island, NY) was used for 50 min at 50°C. The ABI standard 7500 real-time PCR system was used to perform and analyze RT-PCR experiments. Each reaction contained Maxima SYBR Green /ROX MasterMix, 1 mM forward primer and 1 mM reverse primer, cDNA template and molecular grade water. The primers used in this study are shown in **Table S2**. Reactions were performed under the following conditions: 1 cycle at 50°C for 2 min, 1 cycle at 95°C for 10 min, and 45 cycles of 15 sec at 95°C, 1 min at the appropriate annealing temperature and 1 min at 60°C. Samples were then subjected to 15 sec at 95°C, 1 min at 60°C and 30 s at 95°C to allow generation of a dissociation curve and ensure amplification of a specific product. Samples were analyzed by 2% agarose gel electrophoresis.

## Results

### GPs accumulate in a subset of SED DCs following gavage

**Figure 1:**
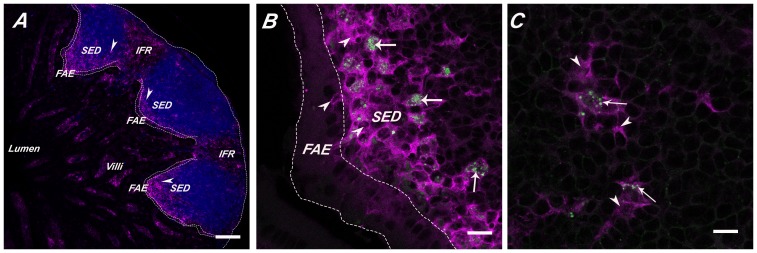
Accumulation of GPs within CD11c^+^ DCs in the SED after oral gavage. GPs were administered to BALB/c mice by gavage, as described in Materials and Methods. PPs were collected 2.5 hr later, cryosectioned, immunostained and viewed by confocal laser scanning microscopy. (**Panel A**) PBS control PP follicles stained in blue with B-cell marker CD45R/B220, CD11c^+^ DCs (magenta;arrowheads). Scale bar is 100 μm. (**Panels B-C**) GPs (green; arrows) were detected within CD11c^+^ DCs (magenta;arrowheads) located within the SED. Scale bar is 50 μm. Abbreviations: FAE, follicle-associated epithelium; SED, sub-epithelial dome; IFR, interfollicular region.

**Figure 2:**
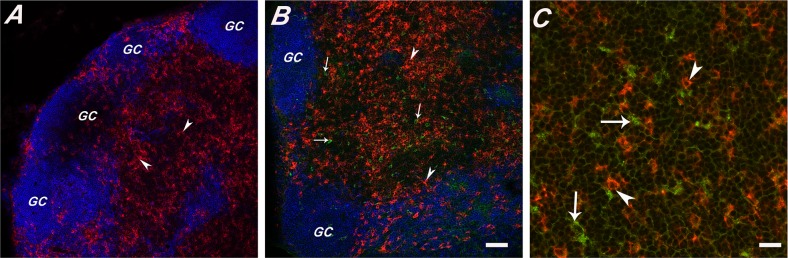
Accumulation of GPs within MLN after oral gavage. MLNs were collected from mice 15(Panels A,B) or 24 hrs (Panel C) after there had been gavaged with GPs. Tissues were cryosectioned, immunostained, and viewed by confocal laser scanning microscopy. (**Panel A**) Cryosections of MLN from vehicle-treated (i.e., PBS) animals. Tissue sections were stained for CD45R/B220 (blue) to detect B cells and CD11c^+^ (red; arrowheads) to detect DCs. (**Panels B-C**) Cryosections of MLN demonstrating that GPs (green; arrows) were regularly associated with CD11c^+^ DCs (red; arrowheads) In panel B the scale bar corresponds to 100 μm, while Panel C it is 50 μm.

**Figure 3:**
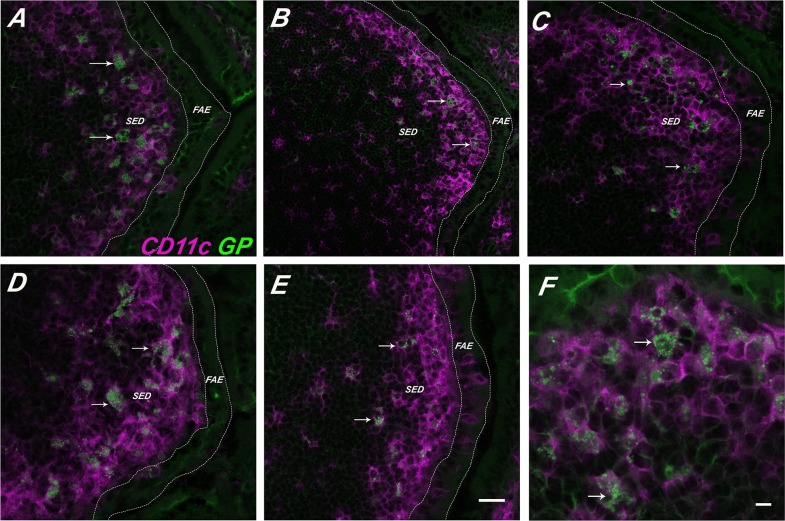
GPs persist within SED DCs. FITC-GPs were administered to BALB/c mice by gavage, as described in Materials and Methods. PPs were collected at indicated time points thereafter and then cryosectioned, immunostained with anti-CD11c antibodies and viewed by confocal laser scanning microscopy. FITC-GPs are shown in green (arrows) and CD11c^+^ DCs in magenta (arrow heads). The panels correspond to the following time points (in hours) (**A**) 2.5; (**B**) 6; (**C**) 24; (**D**) 48; (**E**) 72 hrs. (F) Close up of SED CD11c^+^ DCs from tissues taken at 24 hr. In Panels A-E the scale bar corresponds to 50 μm. In Panel F the scale bar is 20 μm.

In an effort to determine the fate of GPs following intragastric delivery, groups of BALB/c mice were gavaged with 1×10^7^ FITC- or APC-labeled GPs. Twenty-four hours later, the animals were euthanized and segments (proximal, middle and distal) of the small intestine, colon, MLN, spleen, and liver were removed, crysosectioned, and analyzed by confocal microscopy. We observed the accumulation of GPs in the SED regions of PP present throughout the length of the small intestine (**Figure 1**), but not in the intestinal lamina propria (data not shown). We also observed GPs in the MLN (**Figure 2**) but not in the colons, spleens, or livers (**Figure S1**). Within the PP SED region, GPs were associated with DCs and not macrophages, as evidenced by co-localization with CD11c (**Figure 3**) but not CD11b (see below). To determine whether the GPs were actually internalized by PP DCs, and not simply bound to their surfaces, single cell suspensions of PP cells from mice that had been gavaged with FITC-GPs were immunolabeled with CD11c, and then subjected to imaging flow cytometry (ImageStream) analysis (**Figure S2**). By this method, we readily observed FITC-GPs within CD11c^+^ cells, providing evidence that the GPs were in fact internalized by local DCs. We estimate that ∼2% of total PP CD11c^+^ cells had internalized at least one GP.

**Figure 4:**
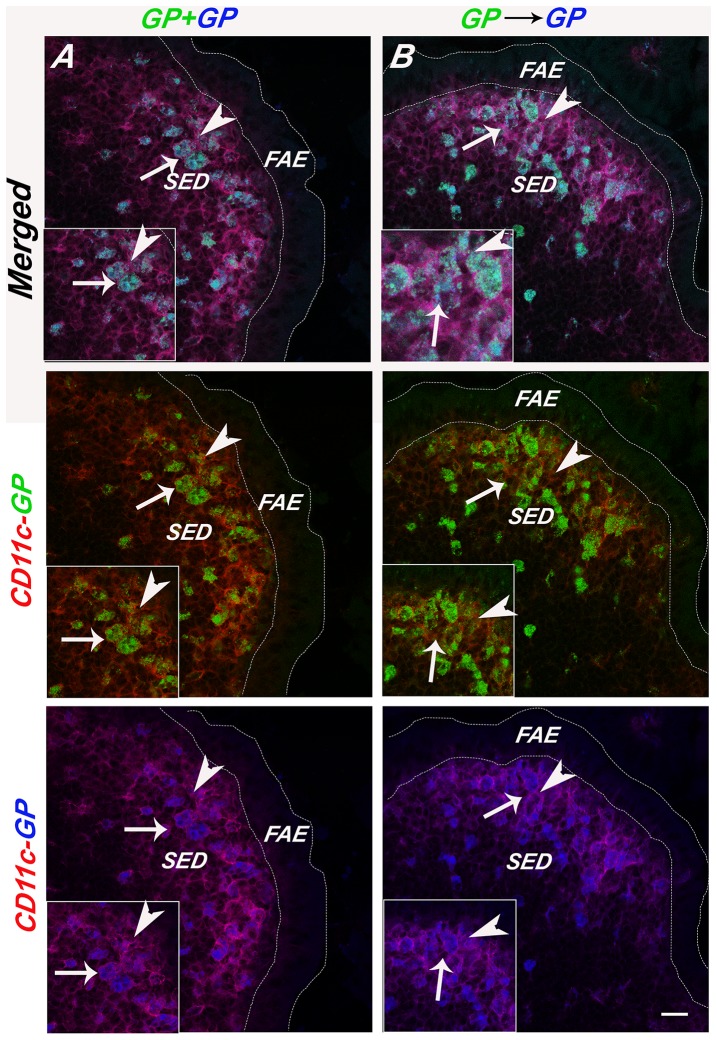
Sampling of differentially labeled GP microparticles by CD11c^+^ DCs. FITC-labeled GPs (green) or APC-labeled GPs (blue) particles were administered to mice by gavage, as described in Materials and Methods. APC-GPs were either (**Panel A**) co-gavaged with FITC-GPs (GP+GP) or (**Panel B**) administered to mice 1 hr after FITC-GPs (GP>GP). Twenty-four hours later PP were collected, cryosectioned, immunostained with PE-labeled CD11c (arrowheads; red) and viewed by confocal laser scanning microscopy. The top pair of panels is a merge of the FITC, APC and PE channels, the middle panels are red and green, and the bottom red and blue, as indicated by the vertical annotation on right. In the left panels, the scale bar corresponds to 100 μm, while the scale bar in the right panel corresponds to 20 μm.

**Figure 5:**
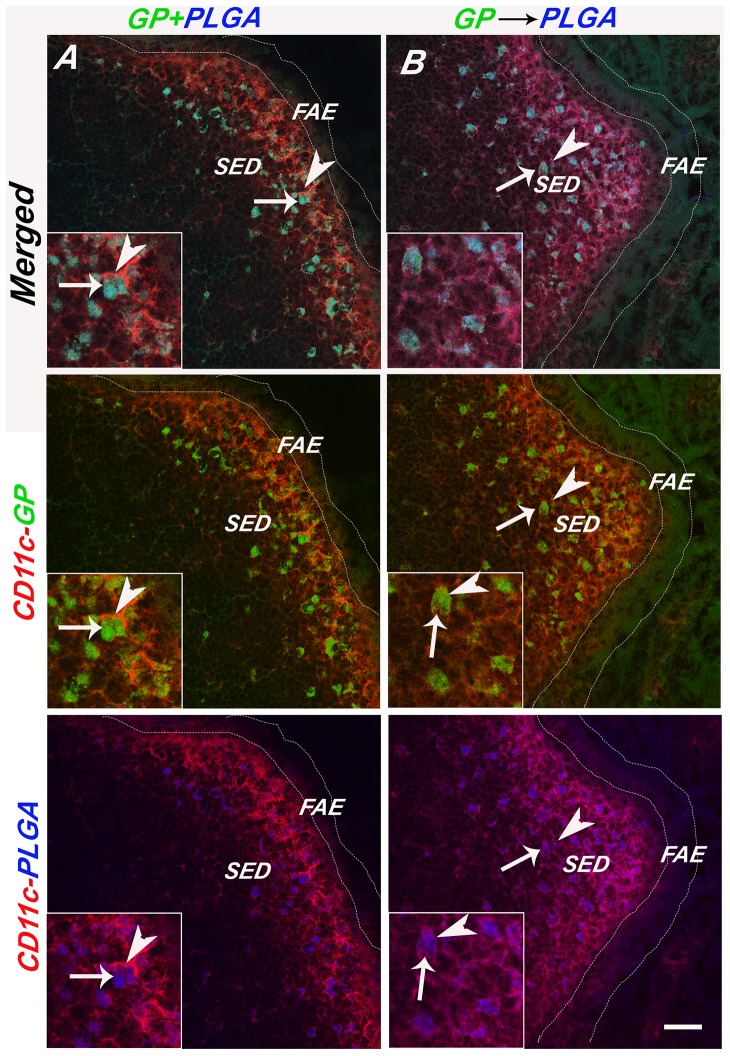
Sampling of GP and PLGA microspheres by SED DCs. PLGA microspheres (blue) were either (**Panel A**) co-gavaged with FITC-GPs (PLGA+GP) or (**Panel B**) administered to mice 1 hr after FITC-GPs (PLGA>GP). Twenty-four hours later PP were collected, cryosectioned, immunostained for CD11c (arrowheads; red) and viewed by confocal laser scanning microscopy. Selected GPs are noted with an arrow. The top pair of panels is a merge of the FITC, APC and PE channels, the middle panels are red and green, and the bottom red and blue, as indicated by the vertical annotation on right. Scale bar corresponds to 100 μm.

Uptake of GPs into the GALT was remarkably fast, as a time course study indicated that GPs were detected in proximal PPs as early as 5 min after gavage (**Figures S3 and S4**). Moreover, the GPs persisted in the SED for at least 72 h (**Figure 3**). The persistence of the GPs within the SED is reminiscent of what others have observed with inert microspheres, rotavirus, and even attenuated strains of Salmonella [24], [25], [26]. In fact, to investigate whether the GPs localize to the same populations of DCs that are involved in sampling inert microparticles, we performed gavage studies in which GPs were mixed 1∶1 with differentially labeled GPs or 0.75 μm in diameter PLGA nanoparticles. PP tissues were collected 24 h later and examined by confocal microscopy. We invariably observed the accumulation of GPs and PLGA nanoparticles (i.e., two different colored GPs or GPs and PLGA) within the same CD11c^+^ cells (**Table 1** **;** **Figure 4**). When GPs and PLGA particles were administered to mice by gavage 1 h apart (e.g., PLGA⇒GPs or GPs⇒PLGA), the particles also localized within the same cells (**Table 1** **;** **Figure 5**), demonstrating that SED DCs retain the capacity sample particulate antigens and do not become quiescent following particle uptake.

**Table 1:** Concomitant and sequential sampling of GPs and PLGA nanoparticles by DN DCs.

GP-GP							GP-PLGA					
Together^a^				1 hr apart			Together			1 hr apart		
	P1	P2	Both	P1	P2	Both	P1	P2	Both	P1	P2	Both
**PP**	65±3	64±6	62±6	43±8	53±3	40±6	39±1	39±0	39±1	47±6	52±5	41±6
**MLN**	90±2	91±2	90±2	43±7	52±4	42±4	70±4	73±2	70±3	35±4	38±2	35±2

a P1, first particle; P2, second particle. Particles were administered to mice by gavage as a 1∶1 mixture (“together”) or serially “1 hr apart”. PP and MLN were collected 24 hr later, cryosectioned and viewed by confocal microscopy. Values indicate the average number (± standard deviation) of particle-containing DCs per PP, based on four mice per group and six sections per mouse.

There are at two major sub-populations of DCs within the SED, the myeloid DCs and the so-called double negative (DN) DCs, which can be distinguished from each other based on relative expression of CD11b and lysozyme M (lysoM) [14], [15], [16], [26]. By immunofluorescence microscopy, the myeloid DCs tend to be intermediate or low for CD11b (CD11b^lo/int^) expression and intermediate or negative for lysoM expression, while the DN DCs are negative for both CD11b and lysoM [16]. Both the myeloid and DN DCs are CD8α^−^. Therefore, to identify which subpopulation(s) of DCs are involved in the sampling of GPs, mice were gavaged with 1×10^7^ GPs and PP were collected and stained for CD8α, CD11b, lysozyme M and CD11c (**Figure S4**). We observed intense CD11b staining in the lamina propria and occasionally in the PP; these cells likely correspond to macrophages based on the fact that they were negative for CD11c. In the PP, the subpopulation of CD11b^lo/int^ CD11c^+^ cells we observed constituted less than 5% of the total DCs in the SED, in accordance with what others have reported [15], [16], [26]. The GPs were generally associated with DCs that were CD11b negative, although we cannot formally exclude the possibility that some GPs were also associated with CD11b^lo^ cells, as the limits of immunostaining did not readily enable us discriminate between the two subpopulations (*i.e.*, CD11b^−^ versus CD11b^lo^). Attempts to use flow cytometry as a means to phenotype the GP-containing DCs were largely inconclusive, as the total yield of GP-positive DCs in the PP DC preparations were insufficient to attain statistically significant counts (data not shown). Attempts to phenotype SED DCs based on lysozyme M expression were also unsuccessful, as we generally observed very few if any lysozyme positive cells within the PP, even though immunostaining was done with the same source of polyclonal rabbit anti-lysozyme antibodies described by Lelouard and colleagues [16]. The reason for this discrepancy is unknown, but may be related to the source of the mouse strains. Therefore, we conclude that GPs are internalized by a subset of CD8α^-^ CD11b^-/lo^ DCs in the SED, even though we cannot definitively ascribe these to the myeloid or DN DC subsets.

### GPs accumulate within a subset of SED DCs characterized by expression of Langerin

Previous studies have suggested that there are multiple subsets of DCs within the mouse PP that can be distinguished from each other by surface expression of CLRs [18], [27]. As little is known about CLR profiles in the PP of BALB/c mice, we used RT-PCR, flow cytometry, and immunostaining to investigate expression of DC-SIGN, SIGN-R1, SIGN-R3, Dectin-1, Dectin 2, Langerin (CD207), and the mannose receptor (MR). RT-PCR and flow cytometric analysis were done with purified PP CD11c^+^ cells obtained by magnetic bead separation, while immunostaining was done on whole PP cryosections.

By RT-PCR, we detected expression of all seven CLRs tested (**Table 2**). By flow cytometry, a fraction of the CD11c^+^ PP DCs stained positive for Dectin-1 (3%), MR (4%), DC-SIGN (7%) and SIGN-R1 (1%) (**Table 2**). By immunofluorescence microscopy, we were unable detect Dectin-1, MR, DC-SIGN, or SIGN-R1 expression in PP DCs, although DC-SIGN and SIGN-R1 staining was evident in the lamina propria (****; data not shown). Since Dectin-1 has been shown to be important in the uptake of GPs in the spleen, we examined the uptake of GPs into SED DCs in Dectin-1^-/-^ mice, as well as available MR^-/-^ and Dectin-1^-/-^/MR^-/-^ double knock-out mice. We found that, as compared to wild type control mice, GP uptake was unchanged with respect to particle localization and GP numbers in Dectin-1^-/-^, MR^-/-^ or the Dectin-1^-/-^/MR^-/-^ double knock-out mice, demonstrating that these receptors are not necessary for GP sampling by PP DCs (**Figure S6**).

**Table 2:** CLR expression in BALB/c PPs.

Marker	CD antigen	Clone	PP-IFM^a^	LP-IFM^a^	FC b	RT-PCR c
DC-SIGN	CD209	MMD3	−	++	7%	+
SIGN-R1	CD209b	22D1	−	−	<1%	+
SIGN-R3	CD209d	−	−	−	n.d.	+
Dectin-1	CLEC-7A	GE2	+	−	3%	+
Dectin-2	CLEC-6A	D2.11E2	−	−	n.d.	+
MR	CD206	MR5D3	−	−	4%	+
Langerin	CD207	RMUL.2	++	−	3%	+

a Positive staining in PPs (PP) or lamina propria (LP) detected by immunofluorescent microscopy (IFM);

b Percent of total PP cells positive for indicated markers, as determined by flow cytometry (FC).

c Detection of indicated marker in isolated PP DCs.

**Figure 6:**
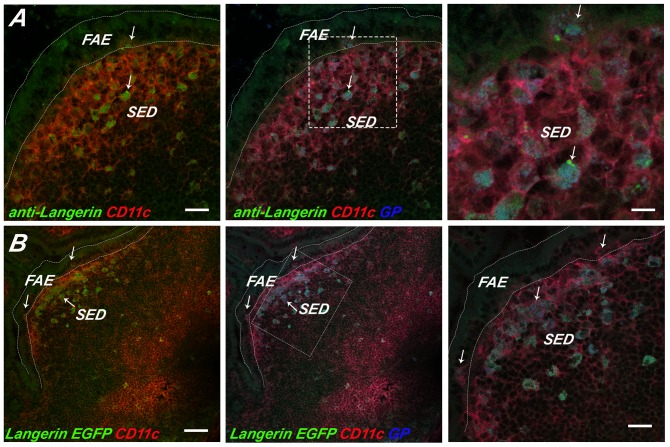
GPs localize within Langerin^+^ SED DCs. APC-labeled GPs (blue) were administered to (**Panel A**) BALB/c or (**Panel B**) Langerin E-GFP-DTR transgenic mice by gavage, as described in Materials and Methods. PP were collected 24 h later, cryosectioned, immunostained and viewed by three color, confocal laser scanning microscopy. (**Panel A**) In panel A, BALB/c PP cryosections were immunostained with anti-Langerin mAb RMUL.2 (green; arrows) and anti-CD11c (red). In Panel B, PP cryosections from Langerin-EGFP-DTR transgenic mice were immunostained with anti-CD11c (red) only. In both panels, the left box shows Langerin (green) and CD11c (red) signals only, the middle box shows Langerin (green), CD11c (red), and the APC-GPs (blue) signals, while the right boxes represent a magnification of the dashed square in the middle box. Scale bars correspond to 100 μm (right and middle) or 50 μm.

Our immunofluorescence and RT-PCR survey did, however, reveal that Langerin was expressed at notable levels in the SED. Langerin is a CLR with specificity for β-D-glucan and that has been shown to mediate the internalization of a wide range of microbial pathogens, including bacteria, viruses and fungi [19], [20], [28], [29], [30]. The population of Langerin^+^ CD11c^+^ DCs in the PP were particularly concentrated in the SED (**Figure 6A**), an observation that is in agreement with what Rochereau and colleagues reported in C57BL/6 mice [18]. By flow cytometric analysis, Langerin positive cells constituted ∼2–3% of total PP cells and 6% of CD11c^+^ PP cells (**Table 2**). We also observed Langerin^+^ cells in the lamina propria, although those cells were likely macrophages as they stained positive for CD11b and not CD11c (**Figure S7**) [31]. As further validation that a Langerin^+^ population of DCs exist in the PP, we examined cryosections of PP from Langerin E-GFP transgenic mice (**Figure 6B**). Indeed, a population of Langerin^+^ of DCs was observed in the SED and were coincident with what we had observed by immunostaining.

As Langerin is known to bind β-glucans and has been shown to recognize fungal particles [19], we next examined whether GPs specifically accumulated within this subset of DCs following delivery to mice by gavage. Specifically, BALB/c or E-GFP transgenic mice were gavaged with 1×10^7^ APC-labeled GPs with or without the addition of PLGA nanoparticles. Twenty-four hours later, the animals were euthanized and PP cryosections were stained with CD11c antibodies and viewed by confocal microscopy. Examination by immunostaining suggest that approximately 80-90% of the GPs localized within Langerin^+^ CD11c^+^ DCs in the BALB/c background (**Figure 6A**). Analysis of Langerin^+^ E-GFP transgenic mice indicated that 72% (±4%) of the GPs resided within Langerin^+^ cells; the remainder of the GPs in the SED were within Langerin^+^ CD11c^+^ cells (**Figure 6B**). Based on these studies, we postulate that Langerin serves as a marker of the unique subset of SED DCs responsible in large part for sampling particular antigens following M cell transepithelial transport.

## Discussion

GPs represent a highly versatile and customizable vaccine delivery platform for a wide range of antigens (*e.g.*, proteins, DNA, siRNA). GPs are also compatible with the forthcoming second generation adjuvants [32]. At this point in time, however, the use of GPs has largely focused on parenteral routes of delivery [2], [3], [4], [5], [6]; only recently have efforts begun to investigate the use of GP technology for the purpose of oral delivery of therapeutics and vaccines [10], [33], [34]. With the goal of using GPs for mucosal delivery of vaccines for biodefense and emerging infectious diseases, we investigated in this study the fate of GPs following oral delivery. In a mouse model, we found that following gavage, GPs rapidly accumulated within with a subset of DCs within the SED regions of PPs. The GPs were detected in PP within minutes after gavage and remained resident in the GALT for at least three days. The same subset of SEDs involved in GP sampling was also responsible for the uptake of PLGA nanospheres, as evidenced by co-localization studies. The most significant finding of this study, however, was the discovery that the SED DC subset involved in GP uptake was invariably positive for Langerin (CD207), a CLR known to recognize glucans and mannose-containing oligosaccharides [19]. While Langerin has been reported to be present on intestinal DC subsets in mice and humans in a variety contexts (as will be discussed below), our study is the first to associate Langerin with a subset of PP DCs involved in sampling of mucosal antigens following transport across the FAE. We speculate that the Langerin^+^ subset of SED DCs is specialized to respond to specific microbial antigens, pathogens [35] and, possibly, even commensal microbes [36].

With respect to the kinetics of uptake and retention within the PP, we found that the GPs were similar to polystyrene and PLGA nanospheres in that, following gavage, they readily accumulated in the SED and remained there for days. While we terminated our studies after three days, Shreedhar and colleagues noted that fluorescent polystyrene particles were detectable in the PP for up to 14 days [26]. Shreedhar also reported that migration of nanoparticle-loaded DCs from the SED to the IFR or B cell follicles could be induced upon exposure of the PP to cholera toxin (a known mucosal adjuvant) or virulent *Salmonella enterica* serovar Typhimurium (*S*.Typhimurium), suggesting that SED DCs remain quiescent until triggered by a “danger signal” or toll-like receptor (TLR) agonist. On the one hand, it is somewhat surprising that GPs themselves were not sufficient to trigger DC emigration from the SED, considering the intrinsic adjuvant properties of β-1,3-D-glucans [7], [8], [9], coupled with the fact that GPs have been shown to induce robust DC activation *in vitro* [3]. On the other hand, considering the local environment, the threshold to trigger SED DCs movement and activation may be quite high, as these cells are likely exposed to a constant stream of luminal antigens, possibly even including fungal components of the microbiota [37]. Indeed, Hopkins and colleagues demonstrated that a strain of *S*.Typhimurium attenuated in its capacity to replicate intracellularly, but replete with TLR agonists (*i.e.*, flagellin, LPS), accumulated and resided within SED DCs for days.

Kelsall and Strober were among the first to describe a population of DCs within the SED that are distinct from DCs elsewhere in the PP [13]. It was subsequently shown that there are in fact two subsets of SED DCs in the mouse PP, the myeloid and the DN DCs, which differ in their levels of CD11b surface expression [14], [15]. Very recently Lelouard and colleagues demonstrated that the myeloid DCs can be further subdivided based on lysozyme M expression [16]. The myeloid DCs were shown to prime naive T cells to secrete high levels of IL-4 and IL-10 and to promote IgA production by naïve B cells [38], while the DN DCs prime T cells for IFN-γ production [15]. While our studies demonstrate unequivocally that GPs accumulate within a subset of SED DCs, we are reluctant at this point to claim whether they are sampled exclusively by the myeloid or DN populations DC that are known to reside just below the FAE [14], [15], [16]. By immunostaining, it was not possible to confidently discriminate between CD11^lo^ and CD11b^-^ expression. By flow cytometry, the number of GP-loaded DCs was generally too low to enable quantitation of CD11b expression. However, the fact that GPs and nanospheres accumulated in the same subset of SED DCs would argue, based on the work by Shreedhar and colleagues, that the GPs likely accumulate in the DN DC subset [26].

As alluded to above, several groups have reported Langerin^+^ DCs in the context of the intestinal mucosa. Chang and colleagues identified the appearance of Langerin^+^ DCs in the intestinal lamina propria of mice that had been subject to vitamin A deficiency[31], while Rochereau and colleagues recently detected Langerin^+^ cells in the PPs of normal, healthy C57BL/6 mice, using acetone fixed cryosections and antibody clone 808E10 [18]. Two recent reports have described Langerin^+^ DCs in the colonic mucosa of humans [39], [40]. Langerin (CD207), a CLR specific for β-glucans, mannose, fucose and N-acetylglucosamines [19], [28] and a well established marker of Langerhan cells, a population of DCs present in the epidermis of the skin [41]. We confirmed and extended Rochereau's findings in that we detected Langerin expression in the PP using both immunofluorescence microscopy and E-GFP transgenic mice. More importantly, we are the first to demonstrate that Langerin marks a subset of PP DCs involved in sampling of mucosal antigens (*i.e.*, GPs and nanopartices) following transport across the FAE. At this point, we can only speculate as to whether Langerin plays a functional role in antigen sampling by SED DCs. As Langerin has been shown to bind and internalize β-glucan particles, it is likely that it also functions in GP uptake *in vivo*. We are currently breeding Langerin knock-out and CD207-EGFP-DTR transgenic mice to test this hypothesis [42].

## Supporting Information

Figure S1 **GP localization in other tissues**. Mice were gavaged with FITC-GPs as indicated in Materials and Methods and sacrificed 24 hr later. The following tissues were collected and screened by confocal microscopy for FITC-GPs: (A) colon, (B) spleen and (C) liver. CD11c^+^ DCs are labeled in red (arrowheads). Abbreviations: villus (V); white pulp (WP); red pulp (RP); marginal zone (MZ). Scale bar is 100 μm.
(TIF)

Figure S2 **Image Stream analysis confirms GPs within CD11c^+^ DCs.** Mice were gavaged with GPs as indicated in Materials and Methods and sacrificed 24 hr later. Single-cell suspensions of total PP cells were subjected to Image Stream analysis. Images reveal CD11c^+^ DCs (red) containing at least one GPs (green). Scale bar is 7 μm.
(TIF)

Figure S3 **GP uptake along the murine small intestine.** The murine small intestine measures about 35 cm and on average contains about 7–9 PP.
(TIF)

Figure S4 **Phenotypic characterization of GP-containing PP DCs.** FITC-labeled GPs were administered to mice by gavage, as described in Materials and Methods. PPs were collected from mice 24 hr later, cryosectioned, immunostained and viewed by confocal laser scanning microscopy. GP appear green in all panels (A–C). (**Panel A**) GP (arrows) do not co-localize with lymphoid CD8α^+^ (red) DCs (arrowheads) (**Panel B**) GP within the SED (green; arrow) do not colocalize with CD11b^+^ (red) myeloid cells (arrowheads) in the SED. Also shown are CD11b^+^ (red) myeloid cells in the lamina propria. (**Panel C**) GP within the SED (green; arrow) are not associated with lysozyme M^+^ DC (red; arrow heads). Fluorescent channels green and red are separated for clarity FAE, follicle-associated epithelium; SED, sub-epithelial dome. Scale bar is 50 μm.
(TIF)

Figure S5 **DC-SIGN, SIGN-R1 and Dectin-1 are not detected in PP follicles.** (A) DC-SIGN (green) arrows is detected in the lamina propria but not in PP follicles. To determine the cell type in lamina propria (LP) that is positive for DC-SIGN we tested CD11b, CD11c and CD45R/B220 magenta (inset) (B) SIGN-R1 (green) was detected in the lamina propria (LP) but not in PP follicles or in SED DCs (magenta; arrow heads). C) Dectin-1 (green) was not detected in the lamina propria or in SED DCs (magenta; arrow heads). Scale bar is 100 μm.
(TIF)

Figure S6 **GP uptake into PP DCs in MR and Dectin-1 knock-out mice.** (A) GPs (green) shown with arrows are found in SED DCs (magenta) in Dectin-1^-/-^ (B) GPs are found in SED DCs in MR^-/-^ (C) Dectin -1^-/-^ / MR^-/-^ also contain GPs in SED DCs. Scale bar is 100 μm.
(TIF)

Figure S7 **Langerin positive cells in PP are not CD11b^+^.** Langerin EGFP (green) transgenic mice were stained with DC marker CD11c (blue) and macrophage marker CD11b(red). Langerin can be seen mostly in PP follicles and in some villi (arrows). Scare bar is 100 μm.
(TIF)

Table S1 **Antibodies used for immunostaining in this study.**
(DOCX)

Table S2 **C-type lectin primer sequences used for RT-PCR.**
(DOCX)
