# Supplementary figures and images for: A Population of Langerin-Positive Dendritic Cells in Murine Peyer's Patches Involved in Sampling β-Glucan Microparticles
Source: PLoS One. 2014 Mar 14;9(3):e91002. doi: 10.1371/journal.pone.0091002 (PMC3954581; doi:10.1371/journal.pone.0091002)

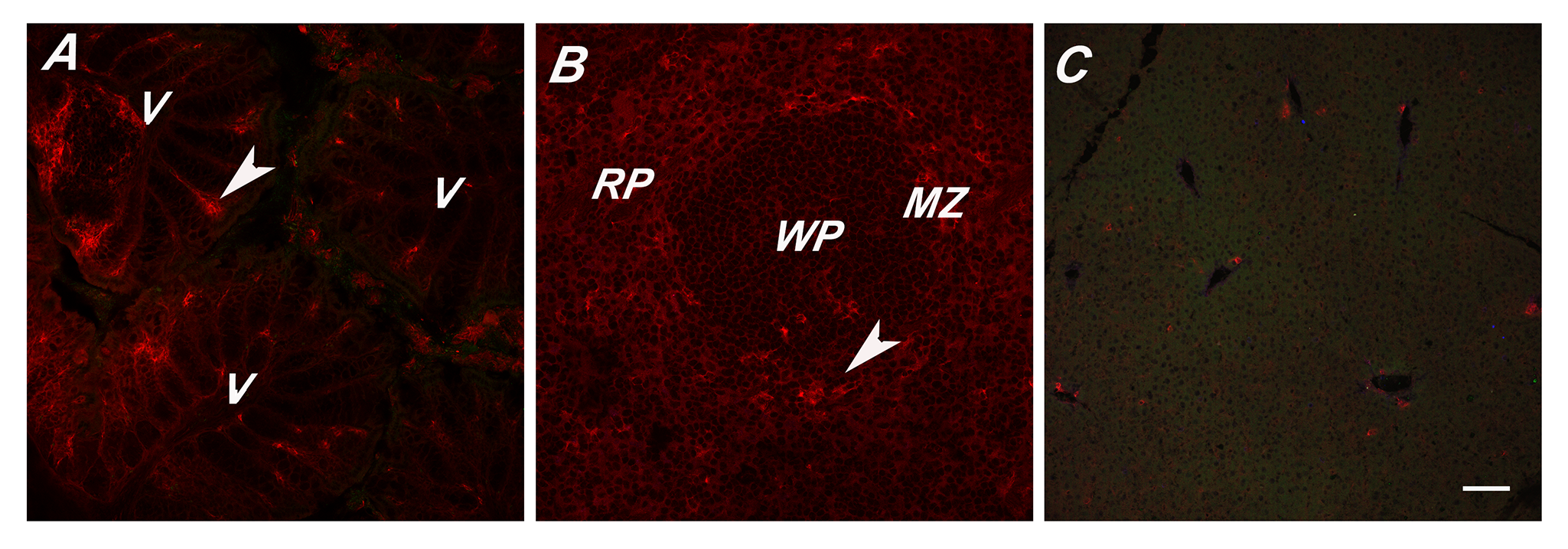

Supplement: Figure S1 — GP localization in other tissues. Mice were gavaged with FITC-GPs as indicated in Materials and Methods and sacrificed 24 hr later. The following tissues were collected and screened by confocal microscopy for FITC-GPs: (A) colon, (B) spleen and (C) liver. CD11c+ DCs are labeled in red (arrowheads). Abbreviations: villus (V); white pulp (WP); red pulp (RP); marginal zone (MZ). Scale bar is 100 μm. (TIF) [file pone.0091002.s001.tif]

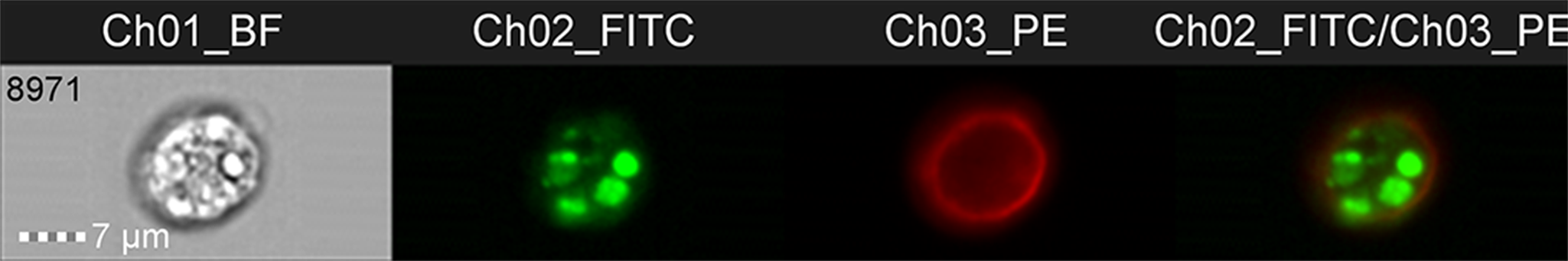

Supplement: Figure S2 — Image Stream analysis confirms GPs within CD11c+ DCs. Mice were gavaged with GPs as indicated in Materials and Methods and sacrificed 24 hr later. Single-cell suspensions of total PP cells were subjected to Image Stream analysis. Images reveal CD11c+ DCs (red) containing at least one GPs (green). Scale bar is 7 μm. (TIF) [file pone.0091002.s002.tif]

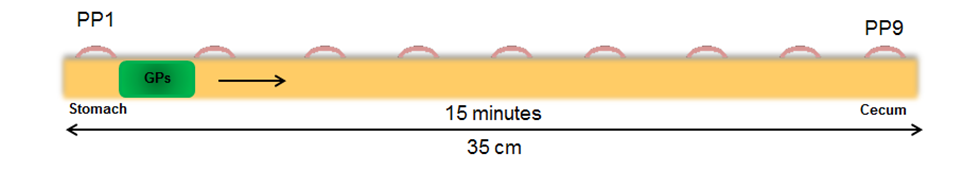

Supplement: Figure S3 — GP uptake along the murine small intestine. The murine small intestine measures about 35 cm and on average contains about 7–9 PP. (TIF) [file pone.0091002.s003.tif]

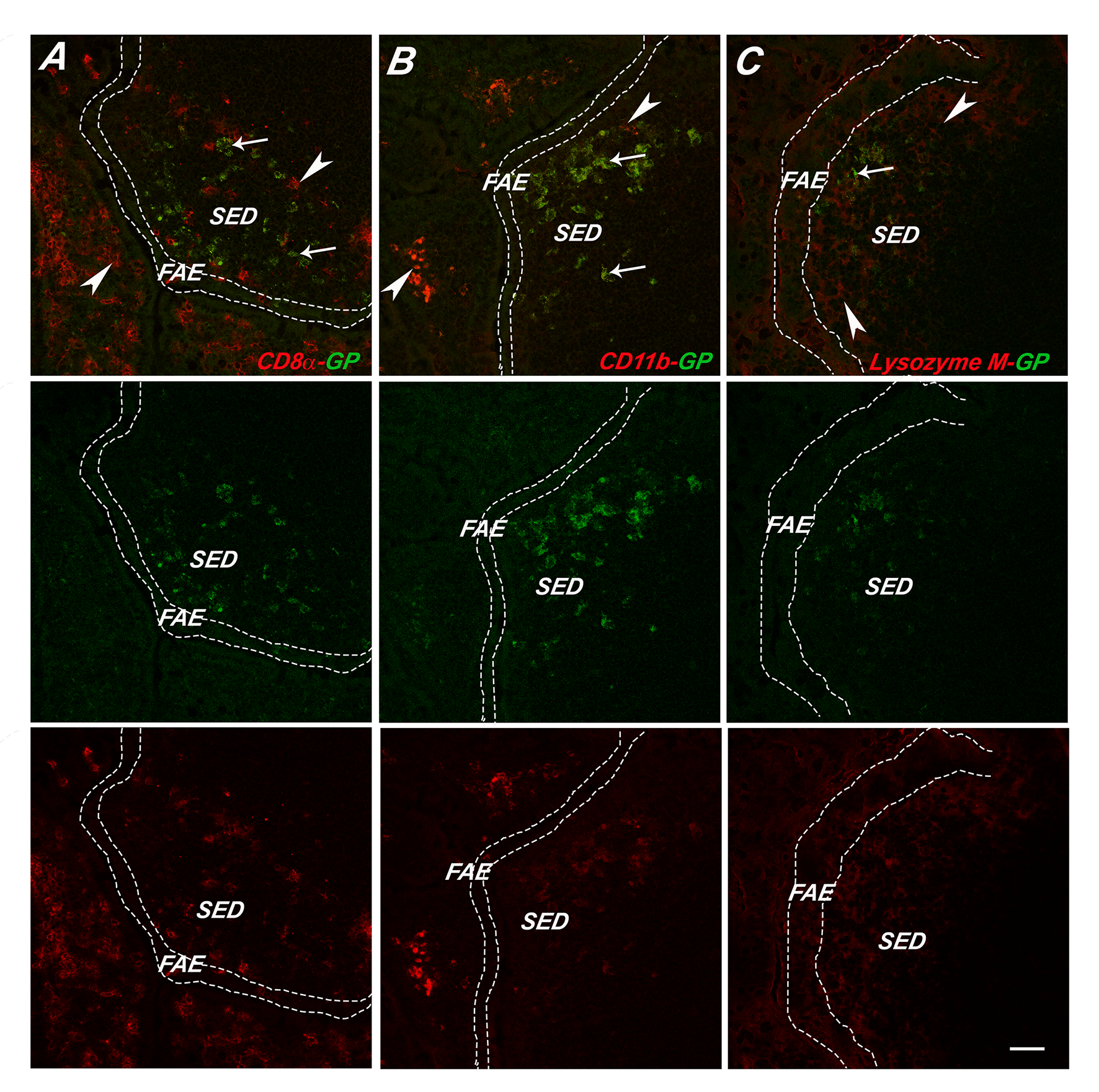

Supplement: Figure S4 — Phenotypic characterization of GP-containing PP DCs. FITC-labeled GPs were administered to mice by gavage, as described in Materials and Methods. PPs were collected from mice 24 hr later, cryosectioned, immunostained and viewed by confocal laser scanning microscopy. GP appear green in all panels (A–C). (Panel A) GP (arrows) do not co-localize with lymphoid CD8α+ (red) DCs (arrowheads) (Panel B) GP within the SED (green; arrow) do not colocalize with CD11b+ (red) myeloid cells (arrowheads) in the SED. Also shown are CD11b+ (red) myeloid cells in the lamina propria. (Panel C) GP within the SED (green; arrow) are not associated with lysozyme M+ DC (red; arrow heads). Fluorescent channels green and red are separated for clarity FAE, follicle-associated epithelium; SED, sub-epithelial dome. Scale bar is 50 μm. (TIF) [file pone.0091002.s004.tif]

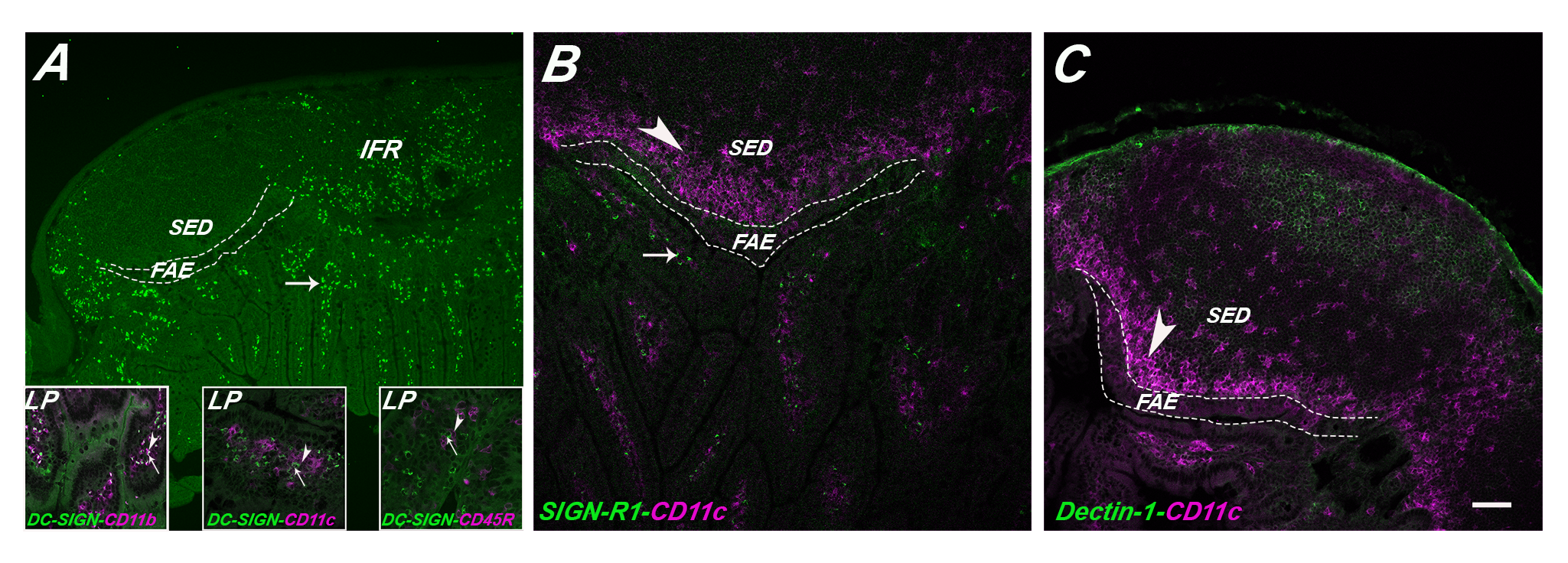

Supplement: Figure S5 — DC-SIGN, SIGN-R1 and Dectin-1 are not detected in PP follicles. (A) DC-SIGN (green) arrows is detected in the lamina propria but not in PP follicles. To determine the cell type in lamina propria (LP) that is positive for DC-SIGN we tested CD11b, CD11c and CD45R/B220 magenta (inset) (B) SIGN-R1 (green) was detected in the lamina propria (LP) but not in PP follicles or in SED DCs (magenta; arrow heads). C) Dectin-1 (green) was not detected in the lamina propria or in SED DCs (magenta; arrow heads). Scale bar is 100 μm. (TIF) [file pone.0091002.s005.tif]

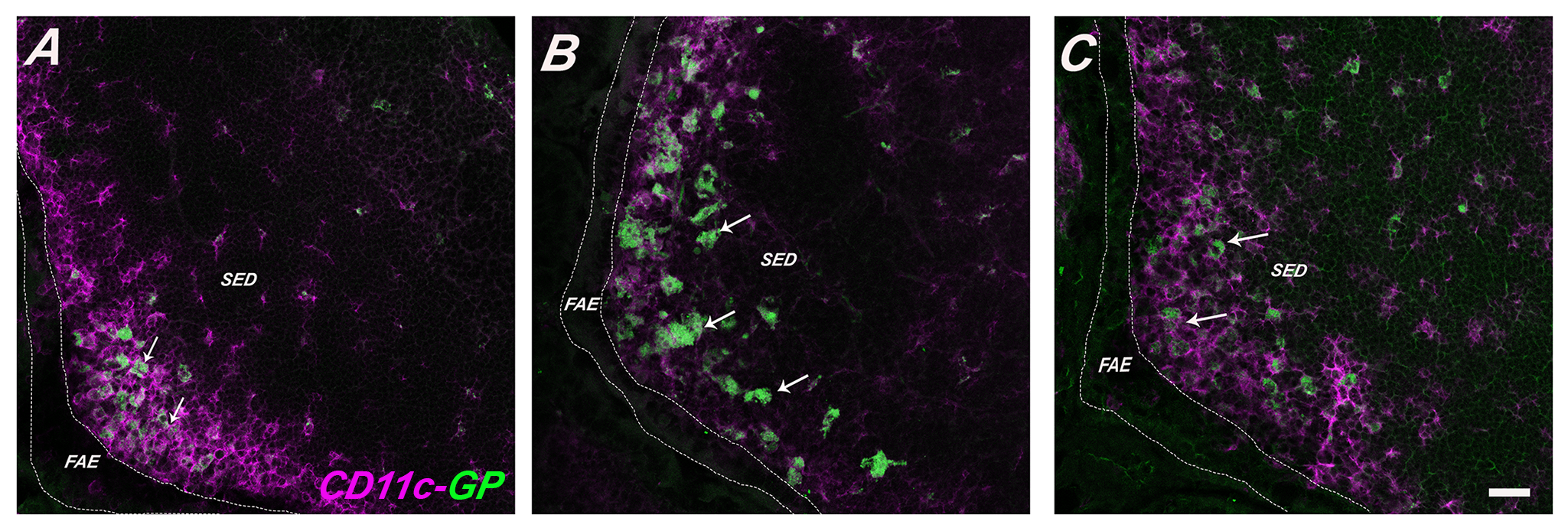

Supplement: Figure S6 — GP uptake into PP DCs in MR and Dectin-1 knock-out mice. (A) GPs (green) shown with arrows are found in SED DCs (magenta) in Dectin-1-/- (B) GPs are found in SED DCs in MR-/- (C) Dectin -1-/- / MR-/- also contain GPs in SED DCs. Scale bar is 100 μm. (TIF) [file pone.0091002.s006.tif]

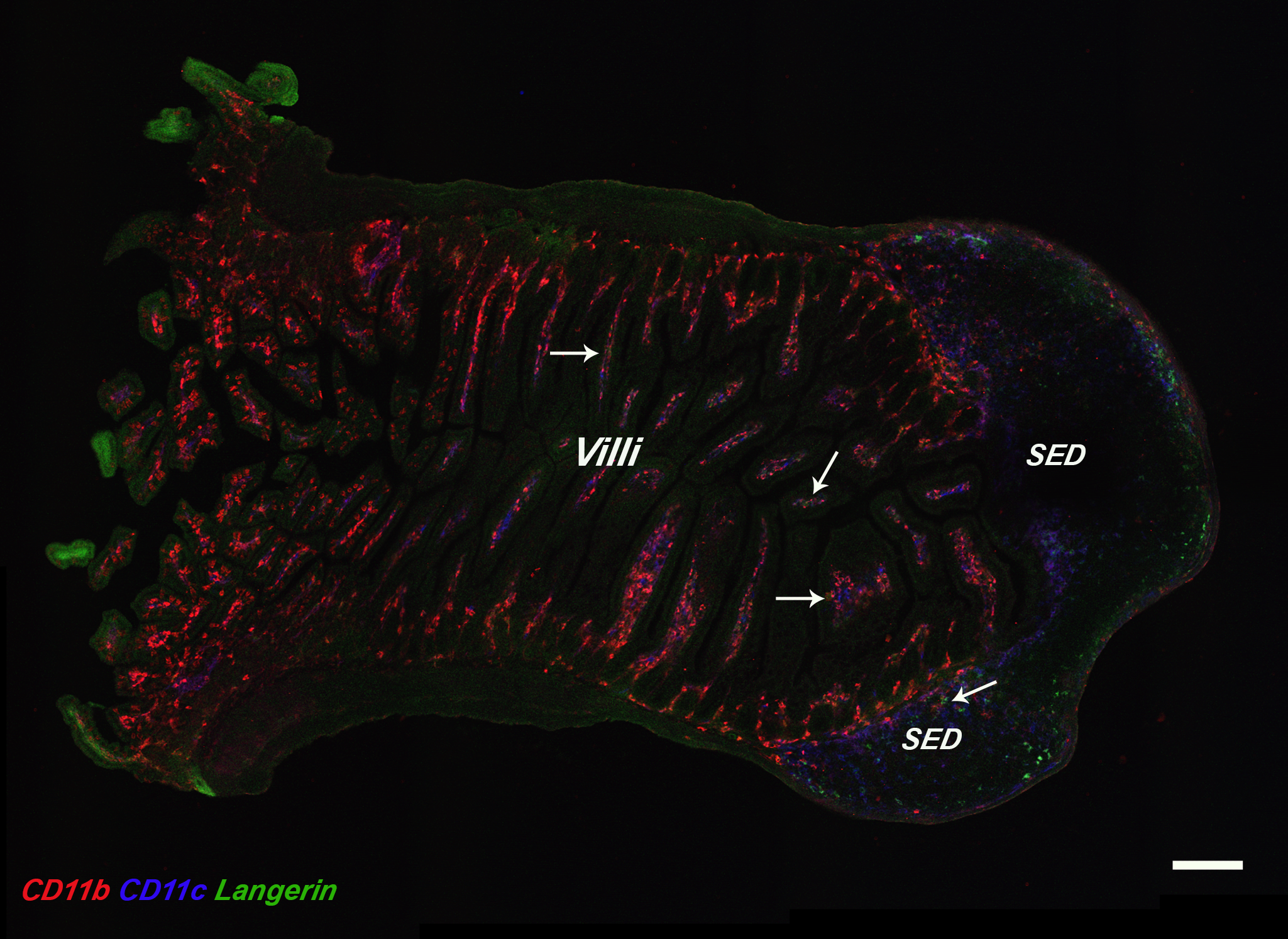

Supplement: Figure S7 — Langerin positive cells in PP are not CD11b+. Langerin EGFP (green) transgenic mice were stained with DC marker CD11c (blue) and macrophage marker CD11b(red). Langerin can be seen mostly in PP follicles and in some villi (arrows). Scare bar is 100 μm. (TIF) [file pone.0091002.s007.tif]
