# Supplementary material for: A Population of Langerin-Positive Dendritic Cells in Murine Peyer's Patches Involved in Sampling β-Glucan Microparticles
Source: PLoS One. 2014 Mar 14;9(3):e91002. doi: 10.1371/journal.pone.0091002 (PMC3954581; doi:10.1371/journal.pone.0091002)
Supplement: Table S1 — Antibodies used for immunostaining in this study. (DOCX) [file pone.0091002.s008.docx]

**Table S1.** Antibodies used for immunostaining in this study.

| **Target Antigen** | **Target Cell or Receptor** | **Source/Reference** |
| --- | --- | --- |
| CD45R | B-cells | BD Biosciences |
| CD11c | Dendritic Cells | eBioscience |
| CD207 | Langerin | eBioscience |
| DC-SIGN | DC-SIGN | C. Park (Rockefeller) |
| CD209b | SIGN-R1 | C. Park (Rockefeller) |
| CLEC-7A | Dectin-1 | eBioscience |
| Dectin-2 | Dectin-2 | AbD Serotec |
| CD206 | MR | AbD Serotec |
| Complement C3 | C3,C3b, iC3b | BioLegend |
| Lysozyme M | Lysozyme | Abcam |

Antibodies used for immunostaining PP sections and for flow cytometry. Source for each antibody is listed.
