# Supplementary material for: A Population of Langerin-Positive Dendritic Cells in Murine Peyer's Patches Involved in Sampling β-Glucan Microparticles
Source: PLoS One. 2014 Mar 14;9(3):e91002. doi: 10.1371/journal.pone.0091002 (PMC3954581; doi:10.1371/journal.pone.0091002)
Supplement: Table S2 — C-type lectin primer sequences used for RT-PCR. (DOCX) [file pone.0091002.s009.docx]

**Table S2.** C-type lectin primer sequences used for RT-PCR

| **Primer Name***^a^* | **Sequence (5’-3’)** | **Annealing Temp °C** | **Reference** |
| --- | --- | --- | --- |
| DC-SIGN F | TGCTGGTTGTCATCCTTGTC | 55 | [[1]](#RANGE!_ENREF_1#RANGE!_ENREF_1) |
| DC-SIGN R | TCTGGGCCACAGAGAAGAAG |  |  |
| Dectin-1 F | AGGCCCTATGAAGAACTACAGACA | 55 | [[2]](#RANGE!_ENREF_2#RANGE!_ENREF_2) |
| Dectin-1 R | TGGCCAGGACAGCATAAGGAA |  |  |
| Dectin-2 F | ACCCCTGACCTTCTGAACATACAC | 55 | [[3]](#RANGE!_ENREF_3#RANGE!_ENREF_3) |
| Dectin-2 R | AAGGGCTCATTCTGTTTG |  |  |
| Langerin F | ACGCACCCCAAAGACCTGGTACAG | 64 | [[4]](#RANGE!_ENREF_4#RANGE!_ENREF_4) |
| Langerin R | AGACACCCTGATATTGGCACAGT |  |  |
| MRC-1F | TCTTTTACGAGAAGTTGGGGTCAG | 64 | [[5]](#RANGE!_ENREF_5#RANGE!_ENREF_5) |
| MRC-1 R | ATCATTCCGTTCACCAGAGGG |  |  |
| SIGN-R1 F | GGCTCCTGCTGATCATTCTT | 52 | [[6]](#RANGE!_ENREF_6#RANGE!_ENREF_6) |
| SIGN-R1 R | CAGTCCCAGGGGCAGAGT |  |  |
| SIGN-R3 F | CTGGGCTTCTGCTGATCATT | 55 | [[6]](#RANGE!_ENREF_6#RANGE!_ENREF_6) |
| SIGN-R3 R | AGTGGTGGAGTTGTGCCAAT |  |  |
| B-actin F | ATGAGGTAGTCTGTCAGGT | 55 | [[7]](#RANGE!_ENREF_7#RANGE!_ENREF_7) |
| B-actin R | ATGGATGACGATATCGTC |  |  |

*^a^*,F and R represent forward and reverse primers.

*^b^*,References cited in Table:

1. Cheong C, Matos I, Choi JH, Schauer JD, Dandamudi DB, Shrestha E, et al. New monoclonal anti-mouse DC-SIGN antibodies reactive with acetone-fixed cells. J Immunol Methods. 2010;360(1-2):66-75. PMCID: 2924951.

2.Park CG, Takahara K, Umemoto E, Yashima Y, Matsubara K, Matsuda Y, et al. Five mouse homologues of the human dendritic cell C-type lectin, DC-SIGN. Int Immunol. 2001;13(10):1283-90.

3.Ariizumi K, Shen GL, Shikano S, Xu S, Ritter R, 3rd, Kumamoto T, et al. Identification of a novel, dendritic cell-associated molecule, dectin-1, by subtractive cDNA cloning. J Biol Chem. 2000;275(26):20157-67.

4.Ariizumi K, Shen GL, Shikano S, Ritter R, 3rd, Zukas P, Edelbaum D, et al. Cloning of a second dendritic cell-associated C-type lectin (dectin-2) and its alternatively spliced isoforms. J Biol Chem. 2000;275(16):11957-63.

5.Takahara K, Omatsu Y, Yashima Y, Maeda Y, Tanaka S, Iyoda T, et al. Identification and expression of mouse Langerin (CD207) in dendritic cells. Int Immunol. 2002;14(5):433-44.

6.Menzies FM, Henriquez FL, Alexander J, Roberts CW. Sequential expression of macrophage anti-microbial/inflammatory and wound healing markers following innate, alternative and classical activation. Clin Exp Immunol. 2010;160(3):369-79. PMCID: 2883107.

7.Lopez-Guerrero DV, Meza-Perez S, Ramirez-Pliego O, Santana-Calderon MA, Espino-Solis P, Gutierrez-Xicotencatl L, et al. Rotavirus infection activates dendritic cells from Peyer's patches in adult mice. J Virol. 2010;84(4):1856-66. PMCID: 2812372.
